# Molar tooth sign with ataxia and see-saw nystagmus (Joubert syndrome)

**DOI:** 10.4103/0972-2327.78057

**Published:** 2011

**Authors:** N. Byju, James Jose, K. Saifudheen, Mohammed Musthafa

**Affiliations:** Department of Neurology, Medical College, Calicut, Kerala, India

## Introduction

A 16-year-old boy presented with complaints of intermittent slurring of speech, occasional swaying of the body, and abnormal eye movements noticed by the relatives, for one month duration. He was born of a consanguineous marriage. Father and mother were third cousins. He had global developmental delay of mile stone and mental retardation. There was no history of similar neurological illness in the family.

On examination, he had moderate mental retardation. External ocular movements were full and see-saw nystagmus was present. Right facial palsy was present. Other cranial nerves were normal. He had mild to moderate truncal ataxia and hypotonia. Other neurological examinations were normal. Patient had two siblings, one brother and one sister. A detailed neurological examination on them did not reveal any abnormality.

**Figure 1:**
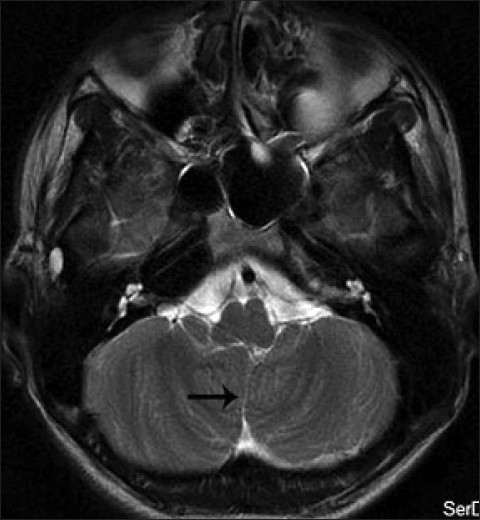
Axial T2 weighted image showing superior vermian dysplasia with agenesis of mid and inferior vermian lobules and apposing cerebellar hemispheres (black arrow)

**Figure 2:**
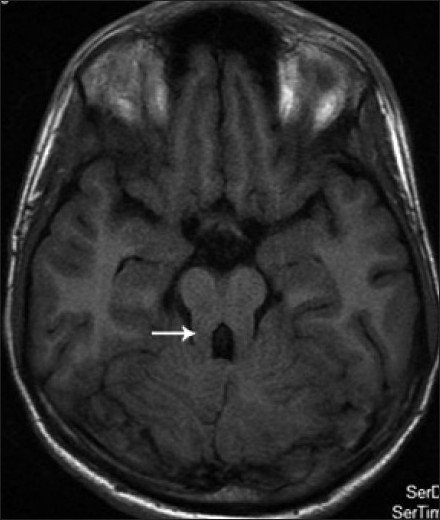
Axial T1 weighted image at the level of mid-brain interpeduncular fossa showing classical molar tooth sign (white arrow)

**Figure 3:**
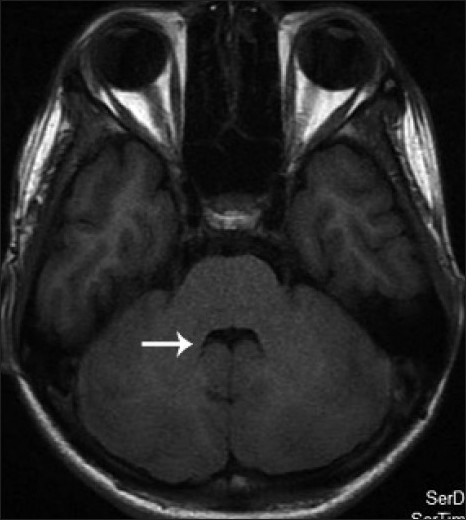
Axial T1 weighted image at the level of the upper pons showing bat-wing morphology of fourth ventricle (white arrow).

**Figure 4:**
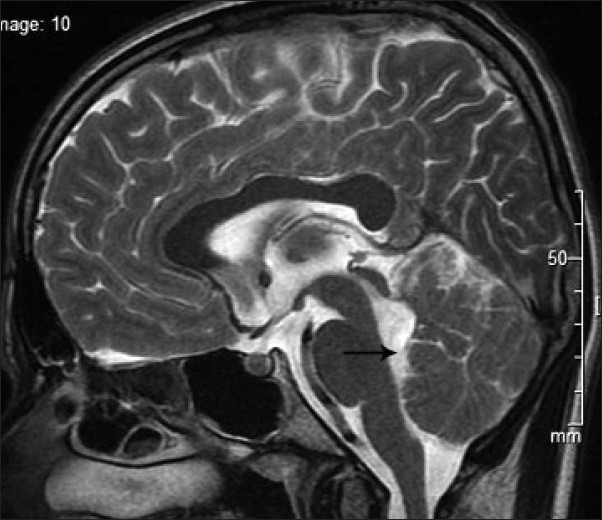
Mid-sagittal T2 weighted image showing a high position of the fourth ventricle and curved roof (black arrow).

His blood and urine routine examinations including renal function and liver function tests were normal. Ultrasonogram of abdomen was normal. His magnetic resonance imaging (MRI) of brain showed vermian agenesis [Figure 1], which is thick and maloriented superior cerebellar peduncles with deep interpeduncular fossa forming the molar tooth appearance [Figure 2]. Fourth ventricle showed a batwing appearance at the level of pons [Figure 3] and the mid sagittal images showed a high position of the ventricles with curved roof [Figure 4]. On the basis of the clinical and MRI findings, a diagnosis of Joubert syndrome was made.

Joubert syndrome was first described by a French Neurologist, Marie Joubert in 1969. It is a rare congenital neurological disorder with autosomal recessive inheritance. In this disease, there is agenesis of cerebellar vermis, malformations of several brain stem nuclei, and dysplasia of the structures of pontomesencephalic junction.

Classical Joubert syndrome is characterized by Molar tooth sign in MRI, hypotonia, developmental delay/mental retardation, oculomotor apraxia, and breathing abnormalities.[1] Other clinical features define subtypes of the disease and they are termed Joubert syndrome and related diseases. These include occipital encephalocele, polymicrogyria, polydactyly, occular coloboma, retinal dystrophy, cystic kidney disease, nephronophthisis, and hepatic fibrosis.[1]

The main oculomotor anomalies described in this syndrome include occulomotor apraxia, decreased smooth pursuit gain and vestibulo-ocular reflexes, hypometric volitional saccades, optic nerve dysplasia, severe visual loss, pendular see-saw nystagmus, gaze-holding nystagmus, and pigmentary changes in the fundus.[2] See-saw nystagmus is an uncommon and interesting eye movement disorder found in this condition characterized by cyclic movement of the eye with conjugate torsional component and disjunctive vertical component. In one-half cycles, one eye will rise and intort and the other eye will fall and extort; and in the next half cycle, the vertical and torsional components are reversed.[2]

The pathognomonic neuroradiological finding in Joubert syndrome and related disorders is the molar tooth sign-with its three components; partial or complete absence of vermice, thickened and elongated superior cerebellar peduncles, and deepened interpeduncular fossa.[3] Lack of normal decussation of the superior cerebellar peduncular fibres leads to enlargement of peduncles, which follow a more horizontal course extending perpendicular to the brainstem between the midbrain and cerebellum.[4] The absence of crossing fibres is also responsible for decreased anteroposterior diameter of the brainstem and deep interpeduncular cistern. The absence of normal vermis creates a midline cleft between two normal appearing cerebellar hemispheres resulting in a characteristic ‘batwing’ appearance of the fourth ventricle on axial MR images. Molar tooth sign is useful in differentiating Joubert syndrome and related diseases from other hindbrain malformations and also aids in delineation of genetic factors.[3]
